# Microbial diversity analysis and screening for novel xylanase enzymes from the sediment of the Lobios Hot Spring in Spain

**DOI:** 10.1038/s41598-019-47637-z

**Published:** 2019-08-01

**Authors:** Kamila Knapik, Manuel Becerra, María-Isabel González-Siso

**Affiliations:** Universidade da Coruña, Grupo EXPRELA, Facultade de Ciencias, Centro de Investigacións Científicas Avanzadas (CICA), A Coruña, Spain

**Keywords:** Metagenomics, Hydrolases

## Abstract

Here, we describe the metagenome composition of a microbial community in a hot spring sediment as well as a sequence-based and function-based screening of the metagenome for identification of novel xylanases. The sediment was collected from the Lobios Hot Spring located in the province of Ourense (Spain). Environmental DNA was extracted and sequenced using Illumina technology, and a total of 3.6 Gbp of clean paired reads was produced. A taxonomic classification that was obtained by comparison to the NCBI protein nr database revealed a dominance of Bacteria (93%), followed by Archaea (6%). The most abundant bacterial phylum was Acidobacteria (25%), while Thaumarchaeota (5%) was the main archaeal phylum. Reads were assembled into contigs. Open reading frames (ORFs) predicted on these contigs were searched by BLAST against the CAZy database to retrieve xylanase encoding ORFs. A metagenomic fosmid library of approximately 150,000 clones was constructed to identify functional genes encoding thermostable xylanase enzymes. Function-based screening revealed a novel xylanase-encoding gene (*XynA3*), which was successfully expressed in *E*. *coli* BL21. The resulting protein (41 kDa), a member of glycoside hydrolase family 11 was purified and biochemically characterized. The highest activity was measured at 80 °C and pH 6.5. The protein was extremely thermostable and showed 94% remaining activity after incubation at 60 °C for 24 h and over 70% remaining activity after incubation at 70 °C for 24 h. Xylanolytic activity of the XynA3 enzyme was stimulated in the presence of β-mercaptoethanol, dithiothreitol and Fe^3+^ ions. HPLC analysis showed that XynA3 hydrolyzes xylan forming xylobiose with lower proportion of xylotriose and xylose. Specific activity of the enzyme was 9080 U/mg for oat arabinoxylan and 5080 U/mg for beechwood xylan, respectively, without cellulase activity.

## Introduction

Xylans, the second most abundant group of polysaccharides in nature and the major component of plant cell walls, are made of a xylose backbone linked by β-1,4-glycosidic bonds and side chains^1^. Xylanases catalyze the hydrolysis of 1,4‐β‐d‐xylosidic linkages in xylan and the hydrolysis products consist of monomers D-xylose and xylooligosaccharides of different sizes^2^. Enzymes degrading xylan are found in microbes, plants, seeds, snails and insects. Glycoside hydrolase families containing enzymes with a demonstrated endo-1,4-β-xylanase activity on xylan have been classified into six GH families 5, 7, 8, 10, 11 and 43 in CAZY database^2^. Among these, families 5, 10 and 11 are known to contain thermostable xylanases^2,3^.

Thermostable xylanases have been recognized as very useful biocatalyst in many industrial applications because they can withstand the harsh conditions of industrial processing without denaturation, like high temperature, alkaline or acidic pretreatment, or the use of solvents. Xylanases are of interest in the food and feed industries, paper and pulp technology, as well as in textile and biofuel production^2^. Hot springs are an interesting source of novel enzymes with potential biotechnological applications because extremely hot environments are inhabited by specialized microorganisms adapted to high temperatures and extreme pHs^4^. Many hot spring-derived xylanases have been recovered from cultured microorganisms^5,6^. However, most bacteria in any given environment cannot be cultured using conventional methods^7^, therefore biotechnological potential from uncultivable hot spring microorganisms remains untapped. The culture-independent metagenomic approaches provide access to the collective nucleic acids from all microorganisms present in an environmental sample^8,9^. Direct sequencing of environmental DNA and homology analysis in comparison with sequences already present in the databases^9^ has led to the identification of many novel enzymes^10^. Another approach is functional metagenomics based on the direct isolation of DNA from environmental samples and generation of metagenomic libraries from the isolated DNA. Screening of the constructed libraries for metabolic activity has led to the identification and characterization of a variety of novel xylanases from soil (Hu and others 2008), compost‐soil^11^, termites^12^ and rumen^13^.

Galicia, a region in the north-west of Spain, is rich in geothermal springs. One of these springs, Lobios, is characterized by high water temperature (76 °C) and alkaline pH (8.2). Our previous study showed that this environment is an attractive source of novel thermostable and alkaline-tolerant enzymes with biotechnological applications^14^. In this study, a sequence-based metagenomic approach was applied to the hot spring sediment sample using the next-generation sequencing to identify candidate genes coding for xylan-degrading enzymes. Moreover, metagenomic DNA sequences were also analyzed to assess the taxonomic composition of the hot spring sediment metagenome and to evaluate the microbial diversity related to GH families of predicted xylanases. Finally, a functional-based metagenomic approach was used to screen for novel xylanase enzymes from constructed metagenomic fosmid library. A thermostable xylanase showing remarkable characteristics for industrial use has been identified, purified and characterized.

## Materials and Methods

### Sample collection and DNA extraction

A sediment sample was collected from an alkaline hot spring located (GPS 41.86113, −8.1062) in Lobios, Ourense, Galicia, Spain, in January 2014. Approximately 400 grams of wet sediment was collected from a borehole into sterile container and transported to the laboratory. The pH of sediment was determined by using pH meter. Briefly, sediment was mixed with distilled water at a ratio (sediment/water) of 1:2.5 and pH was measured after 10 min. Metagenomic DNA was extracted using two independent extractions (20 g of sediment was processed for each extraction) using the PowerMax Soil DNA Isolation kit (Mobio Laboratories Inc.), and a modified protocol, in which cell lysis was achieved by shaking the sediment suspension at 500 rpm in a 65 °C incubator for 45 min.

### Metagenomic sequencing on the Illumina HiSeq platform

Approximately 1 µg of the extracted DNA was sequenced using Pair-end Illumina HiSeq. 1500 platform at Health in Code (A Coruña, Spain). A total of 387,524,268 reads with a read size of 100 bp were generated.

### Read processing and annotation

Paired reads were joined on the overlapping ends using the fastq-join^15^ with a minimum overlap of 8 bp and a maximum difference of 10%. Unpaired reads were discarded. Reads with ambiguous bases (“Ns”), sequence duplicates, minimum quality score of 10 and low-complexity sequences with DUST score < 7 were removed using PRINSEQ (Schmieder and Edwards, 2011). Paired reads were annotated by BLASTX using DIAMOND^16^ against the NCBI protein (nr) database using an e-value 1e-03 and one best match was retained. BLAST output files (in xml format) were imported into MEGAN (MEtaGenome ANalyzer) software (version 5.4.0) to perform taxonomic analysis using a Min score of 50, Top percent value 10%, Min support percent of 0 and Min support of 1^17^. KronaTools-2.7 was used to visualize the BLAST results^18^.

### Read assembly and ORFs prediction

For the assembly, raw reads with ambiguous bases (“Ns”), sequence duplicates, minimum quality score of 25 and low-complexity sequences with DUST score < 7 were removed using PRINSEQ.^19^. Filtered reads were then assembled using IDBA-UD version 1.0.9^20^. The putative Open Reading Frames (ORFs) were predicted from contigs using MetaGeneMark Heuristic Approach^21^.

### Xylanase sequences in metagenomic contigs

CAZYdb (Carbohydrate-Active enZYmes Database) (updated on 20 July 2017) was downloaded from dbCAN^22,23^ (http://csbl.bmb.uga.edu/dbCAN/download.php). The predicted ORFs were used for BLASTP (with e-value of 1e-5 and minimum bit-score of 50) search against the CAZy database using BLAST + (version 2.6.0) for identification of carbohydrate-active enzymes. Sequences classified within the glycosyl hydrolase (GH) families 5, 7, 8, 10, 11 and 43, known to contain enzymes with endo-1,4-β-xylanase activity^2^, were retrieved and further annotated using BLASTP against NCBI nr database.

### Sequence deposition

The raw sequencing reads and the assembled metagenome dataset have been deposited at the NCBI Short Read Archive under BioProject ID PRJNA540576 and accession number SRR9001935. Identified xylanase XynA3 sequence were submitted to GenBank under accession number MK878879.

### Metagenomic library construction

Metagenomic library construction was performed using the CopyControl™ HTP Fosmid Library Production Kit (Epicentre) and the pCT3FK fosmid vector^24^. Approximately 6 µg of high-molecular-weight (HMW) DNA was end-repaired and separated on 1% low melting point agarose gel electrophoresis for 12 h at 40 V. DNA fragments with size ranging from 30 to 40 kb were isolated and recovered from the gel with GELase (Epicentre Technologies). A fosmid library of purified end-repaired 30–40 kb metagenomic DNA was constructed using the CopyControl Fosmid Library Production kit (Epicentre Biotechnologies). Approximately 300 ng of DNA was ligated to 500 ng of the linearized fosmid pCT3FK vector, packaged into the lambda phage and used to infect *E*. *coli* EPI300-T1^R^ cells. The library size was determined by dilution titering on LB (Luria-Bertani) agar plates containing 12.5 μg/ml chloramphenicol (LB/Cm). The resulting fosmid library was plated onto 76 LB/Cm plates at a density of ≈2000 bacterial clones per plate. Colonies from each plate were resuspended in 2 ml of LB containing 20% glycerol and a 150 μl aliquot was transferred to a 96-well plate; each well contained ≈2000 independent clones. The library was stored at −80 °C. The average DNA insert size was estimated by isolation and purification of 20 randomly chosen fosmids.

### Functional screening

20 µl aliquot from metagenomic fosmid library was transferred to a new 96-well plate containing 130 µl of liquid LB/Cm and incubated overnight at 37 °C. Subsequently, the overnight culture was replica plated using a 48-pin replicator stamp onto LB/Cm plates containing 0.02% arabinose and 0.05% insoluble AZCL-HE-xylan (Megazyme) (powder has been transferred in 500 µl of absolute ethanol and added into the medium after autoclaving)^25,26^. The plates were incubated at 37 °C for 7 days. Xylanase activity was identified by the formation of a blue halo around the stamped colonies. The fosmid that showed xylanolitic activity was isolated, digested with DraI and PvuII restriction enzymes and sub-cloned into pJET1.2. The positive sub-clone was sequenced using primer walking and the ORF of the putative xylanase gene was identified using ORFfinder available at https://www.ncbi.nlm.nih.gov/orffinder/.

### XynA3 sequence analysis

The nucleotide sequence of the identified *XynA3* gene was searched against the NCBI nr database using BLASTX and protein sequences of top 7 BLAST hits were retrieved and used to construct a multiple sequence alignment using Clustal Omega^27^. Conserved regions were determined as described previously^28^. The theoretical p*I* (isoelectric point) and Mw (molecular weight) molecular mass of the XynA3 protein sequence was determined using ExPASy’s Compute pI/mw tool^29^. A potential ribosome-binding site was identified by visual inspection. In order to identify longer sequence in the sequenced hot spring sediment metagenome that matched the identified xylanase, the nucleotide sequence of *XynA3* was searched against the metagenomic reads, ORFs and contigs using BLASTN and e-value of 1e-3.

### Cloning, expression and purification of XynA3

The ORF of the putative xylanase gene was PCR amplified with the following primers: XynA3F 5′-AAAATTCATATGAAAAACAGAAAAGGATGGCTG- 3′ and XynA3R 5′-ATTATTGTCTCGAGTTTGATCTCCAGATAATCGAG-3′. The underlined bases indicate NdeI and XhoI restriction sites incorporated into primers. The purified PCR products were digested with NdeI and XhoI and cloned into pET21a expression vector digested with the same restriction enzymes. The resulting recombinant plasmid (pET21a-xynA3) was transformed into *E*. *coli* XL1-Blue and then BL21. Transformed BL21 cells were grown in four flasks containing 1 L of LB medium with ampicillin (100 µg/ml) at 37 °C until an OD600 nm of 0.6–0.8 was reached. The cells were induced by addition of IPTG (isopropyl β-D-1-thiogalactopyranoside) to a final concentration 0.2 mM, and the flasks were further incubated at 37 °C for 2 h. Induced cells were harvested by centrifugation, resuspended in 30 ml of binding buffer (150 mM NaCl, 50 mM Tris-HCl, pH 7.3) and put to −80 °C. Subsequently, the cells were sonicated on ice at 100% amplitude for 15 min consisting of 2 sec pulse-on and 8 sec pulse-off intervals. Insoluble material was removed by centrifugation (20 minutes, 14,000 rpm, 4 °C) and filtration through a 0.45 um syringe filter. Clarified lysate was passed through a 5 ml HiTrap Q HP column (GE Healthcare Life Sciences) that had been equilibrated with the same binding buffer. The sample was eluted with an elution buffer (150 mM NaCl, 500 mM imidazole, 50 mM Tris-HCl, pH 7.3), with a linear imidazole gradient of 30−500 mM at a flow rate of 3.5 ml/min. The eluted fractions showing xylanase activity were combined, concentrated, and further purified by anion exchange using a 5 ml HiTrap SP HP column (GE Healthcare Life Sciences) with a binding buffer composed of 30 mM NaCl, 5 mM β-Mercaptoethanol, 10 mM Tris-HCl, pH 6.8, and eluted with a linear gradient of 0–500 mM NaCl in 5 mM β-Mercaptoethanol, and 50 mM Tris-HCl, pH 6.8. Eluted fractions were pooled, and the proteins were exchanged into a protein storage buffer (50 mM Tris-HCl, 150 mM NaCl, pH 7.3) using Amicon Ultra-15 centrifugal filter unit with Ultracel-30 membrane (Millipore) down to ~1 ml and stored in 100 µl aliquots at −80 °C until use. All purification steps were performed using an ÄKTA Prime purification system (GE Healthcare Life Sciences). SDS-PAGE was used to analyze the purity and the molecular weight of the purified protein. The protein concentration was analyzed using Eppendorf BioPhotometer D30 and Bradford assay.

### Enzyme assays

Enzyme activity was measured using dinitrosalicylic acid (DNS) assay^30^ with xylose (Sigma) as the standard. The composition of DNS reagent was as follows: 8 g of sodium hydroxide, 5 g of 3,5-dinitrosalicylic acid, 150 g of potassium sodium tartrate in 500 ml dH_2_O. The xylanase activity assay was carried out using 1% arabinoxylan (Megazyme) substrate in citrate-phosphate (McIlvaine) buffer^31^. The pH values for buffer solutions were adjusted at room temperature. The optimum pH for xylanase activity was obtained by assaying the purified enzyme at different pH (3.0–8.0) using citrate-phosphate buffer at 60 °C. The optimum temperature was determined by measuring the purified enzyme activity at different temperatures (40–100 °C) at pH 6.5. The assay consisted of 20 μl of appropriately diluted enzyme pre-heated for 30 sec and mixed with 180 μl of pre-heated for 2 min 1% arabinoxylan or beechwood xylan and incubated at respective temperature for 10 min. To stop the reaction, 300 μl DNS was added to the solution, and the mixture was immediately boiled for 5 min, then cooled on ice for 5 min. The absorbance was determined spectrophotometrically at 540 nm. Cellulolytic activity was tested on carboxymethyl cellulose (SIGMA) and AZCL-HE-cellulose (Megazyme). The thermostability of the enzyme was examined by incubating the enzyme in citrate-phosphate buffer (pH 6.5) at 60 °C, 70 °C and 80 °C for 24 h, respectively. The aliquots were withdrawn at different time intervals (10 min, 30 min, 1 h, 2 h, 3 h, 4 h, and 24 h) and the residual activities were measured by the method as described above. One unit of enzyme activity (U) was defined as the amount of the enzyme that will produce 1 μmol of reducing sugar (measured as xylose) from xylan per minute at pH 6.5 at 80 °C.

### Effect of metal ions on xylanase activity

Effects of metal ions and other chemicals on xylanase activity was determined using 1 mM and 10 mM solutions of CaCl_2_, FeCl_3_, CuSO_4_, MnCl_2_, MgCl_2_, or KCl and EDTA, and 0.1% and 1% solutions of SDS, 2-Mercaptoethanol, DTT, N-Bromosuccinimide, Tween 20, Tritone X-100. The xylanase activity was assayed using the DNS method described above. Reaction mixture without any additive was considered as the control and its xylanase activity was designated as 100%.

### Xylose and xylooligosaccharide quantification

The 2% beechwood xylan solution in citrate-phosphate buffer (pH 6.5) was incubated with 20 U of purified XynA3 at 60 °C for 24 h and boiled for 5 min. Samples and standard XOs (xylose, xylobiose and xylotriose) were analysed using a Waters HPLC at 90 °C using a Sugar-Pak^TM^ I column and refractive index detector (Waters 2414). Distilled water was used as the mobile phase with a flow rate of 0.5 ml/min, and injection volume of 15 µl and retention time 14 min.

### Homology modelling

The models of XynA3 and 1,4-beta-xylanase from *Paenibacillaceae* bacterium JTherm (GenBank accession number: PDO11710) were made with the fully automated protein structure homology-modelling server Swiss-Model (https://swissmodel.expasy.org/)^32^ and the figures were generated with PyMOL (The PyMOL Molecular Graphics System, Version 2.1.1 Schrödinger, LLC).

## Results and Discussion

### Taxonomic classification of the sediment prokaryotic community

**Figure 1:**
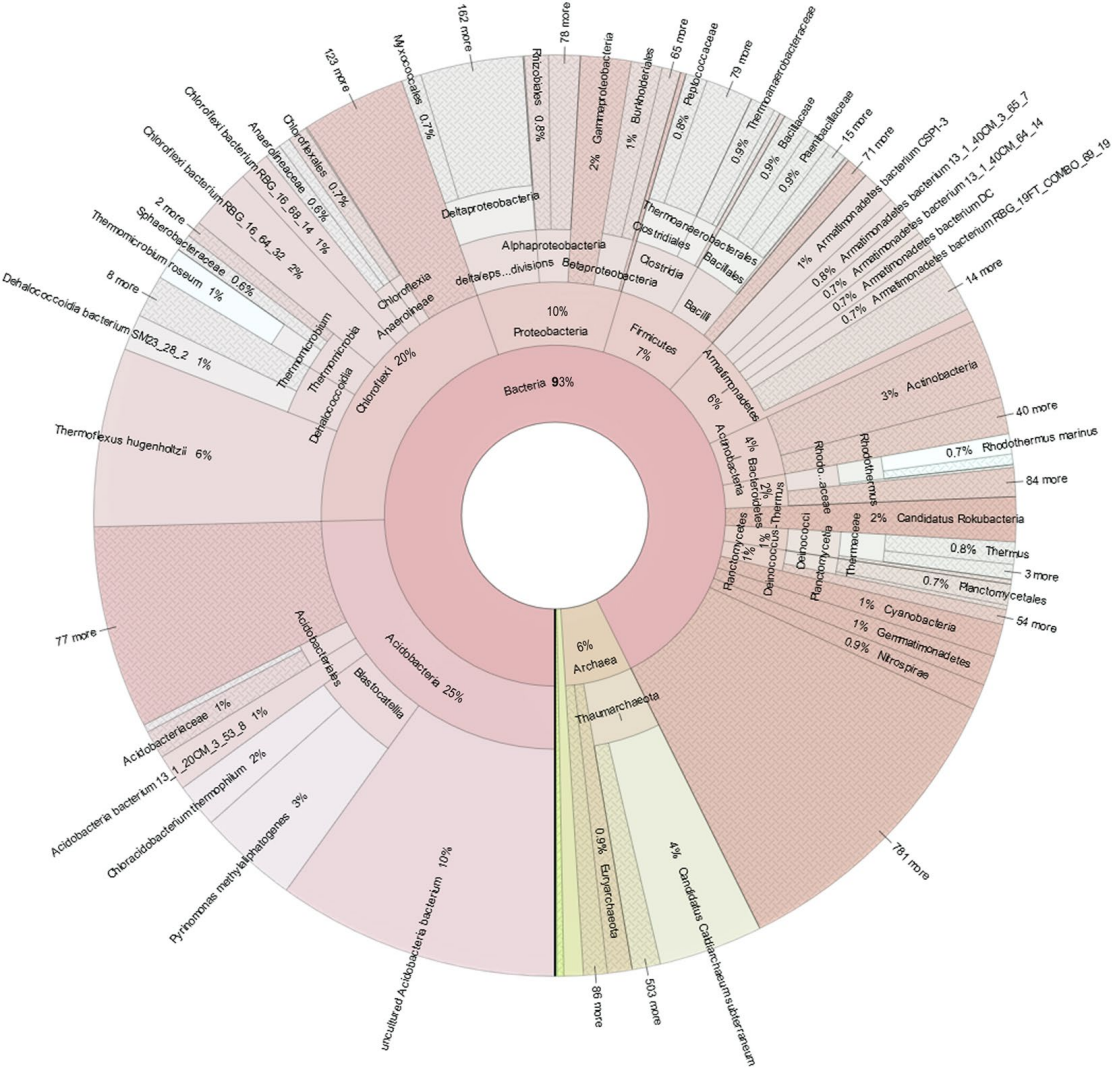
Krona chart representation of taxonomic classification of the hot spring sediment metagenome.

A sediment sample was collected from the Lobios Hot Spring in January 2014. The hot spring sediment pH was acidic (pH 5.9), in contrast to the alkaline pH of water (pH 8.2). The water temperature of this hot spring was 76 °C^14^. The Illumina HiSeq sequencing of the environmental DNA generated 38.8 Gbp of sequence reads. Pairs of overlapping fragments were combined and unpaired reads were discarded. After the quality control, the remaining reads (21,681,238 reads with an average read length of 165 bp and total length of 3.6 Gbp) were used for phylogenetic annotation using DIAMOND against the nr protein database using BLASTX. Taxonomy read counts were exported from MEGAN (File 1 in E-supplementary data) and visualized using Krona (Fig. 1). Of a total of 21.7 M reads, 62% were annotated and classified mainly within the Bacteria and Archaea domains. Bacteria were the most abundant prokaryotic domain, constituting 93% of all annotated reads, whereas archaeal sequences represented only 6% of all annotated reads. The phylum Acidobacteria (25%) was the most abundant among the annotated reads. This is in agreement with culture independent studies which showed that Acidobacteria are widespread in soil, sediments and hot spring environments^33^. The majority of the sequences that fell into that phylum were similar to sequences identified using other metagenomic studies, to the uncultured Acidobacteria bacterium found in a water stream in Japan^34^ and environmental Acidobacteria present in soil samples^35^. This may be due to the fact that Acidobacteria are rarely cultured therefore cultured isolates may be underrepresented in the database^36^. The closest cultured representatives were *Pyrinomonas methylaliphatogenes* and *Chloracidobacterium thermophilum*, bacteria found in geothermal soils and hot springs^37,38^. Second most abundant phylum was Chloroflexi (20%). This phylum was mainly represented by uncultured Chloroflexi bacteria from sediment metagenomic surveys^39,40^ and non-photosynthetic *Thermomicrobium roseum* isolated from a hot spring^41^. Remaining sequences were affiliated with other phyla: Proteobacteria (10%), Firmicutes (7%), Armatimonadetes (6%), Actinobacteria (4%), Bacteroidetes (2%), Deinococcus-Thermus (1%), Planctomycetes (1%), Cyanobacteria (1%), Gemmatimonadetes (1%) and other with abundance less than 1%. Archaeal reads (6%) showed that almost all fell into the phylum Thaumarchaeota (5%), which was predominantly affiliated with Candidatus *Caldiarchaeum subterraneum* (4%) previously isolated from the geothermal water^42^. The most abundant phyla identified in the sediment sample of the Lobios Hot Spring were also observed in the water sample from the same hot spring as determined in our previous study^14^, however the relative abundances of these taxa was different. In the water, sequences related to Deinococcus-Thermus were the most abundant (21%), whereas in the sediment sample their abundance was low (1%). Acidobacteria (25%) and Chloroflexi (20%) were the most abundant in the sediment, and their proportions in the water were less abundant (9% and 7%, respectively).

### Identification of xylan-degrading enzymes by sequence-based approach

**Figure 2:**
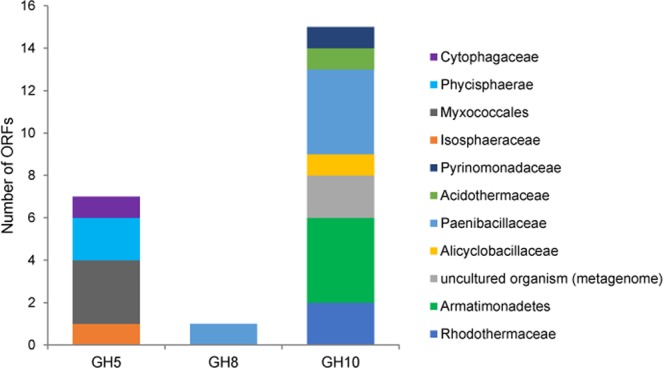
Taxonomic classification of endo‐1,4‐β‐xylanases found in the hot spring sediment metagenome belonging to glycosyl hydrolase (GH) family 5, 8 and 10.

Reads were assembled into 117,315 contigs (totaling 147.8 Mbp). The ORFs (216,305) predicted from these contigs were queried against the CAZy database using a BLAST search, leading to the identification of 15,863 ORFs with significant BLAST hits (E value < 10^−5^). Most abundant CAZy-associated genes identified in the hot spring metagenome were related to glycosyl transferase families GT2 (2,955 ORFs) and GT4 (2,331 ORFs). These large families include diverse proteins involved in biosynthesis of cellulose, chitin, sucrose and N‐glycosylation. These families were also the most abundant in the microbial community decomposing poplar wood chips^43^. Twenty-three putative xylanase-encoding ORFs were identified and classified within the family of GH5 (7 ORFs), GH8 (1 ORF) and GH10 (15 ORFs) (Fig. 2). Five of them were complete or nearly complete. Annotation of these ORFs using the NCBI nr database revealed that they were most similar to bacteria belonging to *Paenibacillaceae* family (5 ORFs), *Armatimonadetes* (4 ORFs) and *Myxococcales* (3 ORFs) (Fig. 2), proteins derived from a hot spring, sediment, compost and soil environment^40,44,45^. The number of putative ORFs involved in degradation of xylan found in this study was lower than that detected in metagenomes from samples with high lignocellulose-degrading ability, such as lignocellulosic biomass microbiota^10^, crop-eating snail microbiome^46^ and yak rumen microbiome^47^. These findings are not surprising as hot spring environment does not generally contain high plant biomas.

### Identification of xylan-degrading enzymes by function-based approach

**Figure 3:**
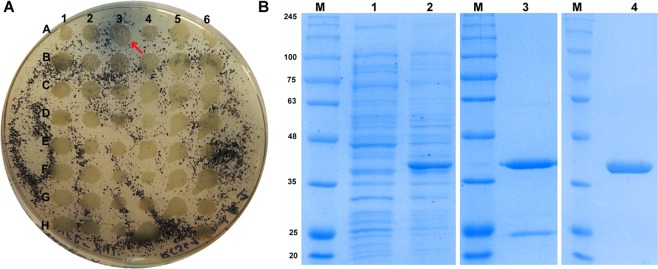
Xylanase identification and purification. (**A**) LB plate containing insoluble AZCL-xylan. The positive stamped clone is indicated by a red arrow. (**B**) SDS-PAGE analysis of recombinant xylanase XynA3 purified from *E*. *coli* (pET21a-xynA3). Lane 1, crude extract; lane 2, crude extract induced with IPTG; lane 3, after Ni-NTA affinity chromatography; lane 4, after ion exchange chromatography; M, NZYcolour Protein Marker II (NZYTech).

A metagenomic fosmid library containing approximately 150,000 *E*. *coli* clones, was constructed from microbial DNA extracted from a hot spring sediment sample. The insert size ranged from 22 kb to 59 kb (average size of 43 kb), covering approximately 6.5 Gbp of DNA in the library. To facilitate expression screening and positive clone recovery efforts, the library was arrayed into 76 wells of 96-well plate at a density of 2,000 clones per well. The library subsequently was screened for xylan active enzymes. Functional screening using azurine-cross linked (AZCL) xylan as a substrate resulted in the detection of a fosmid, named pCT3FK-XynA3, forming a blue halo on the indicator plate (Fig. 3A). The positive hit rate from hot spring sediment was much lower in comparison with rumen metagenome known to have high lignocellulose-degrading ability (52 positive clones per 14,000 fosmid clones screened)^48^.

### Characterization of *XynA3* gene

**Figure 4:**
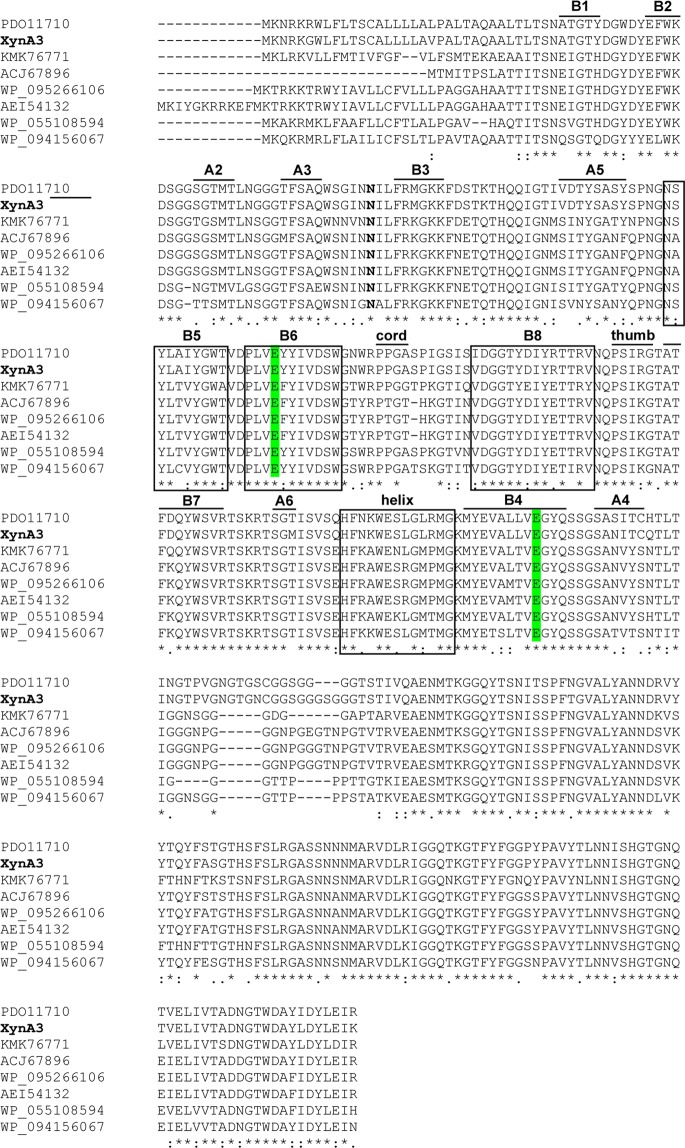
Alignment of the predicted amino acid sequence of XynA3 and its closest relatives from the GenBank databases. The closest sequences shown are: 1,4-beta-xylanase from *Paenibacillaceae* bacterium JTherm derived from compost metagenome (PDO11710); *Paenibacillus ihumii* (WP_055108594); *Paenibacillus kribbensis* (WP_094156067); *Bacillus pseudalcaliphilus* (KMK76771); *Paenibacillus campinasensis* (WP_095266106 and AEI54132) and synthetic construct (ACJ67896). Gaps are indicated by dashes. Asterisks indicate identical amino acids. The black boxes indicate conserved regions and the secondary structure elements are selected by black lines. Asparagine (N) present in alkaline xylanases is shown in bold. The glutamic acid residues corresponding to Glu 122 and 212 of XynA3, essential for the catalytic activity, are highlighted in green.

The DNA insert of fosmid pCT3FK-XynA3 was partially digested with DraI and PvuII and subcloned. One subclone expressing xylanase activity was sequenced and an ORF encoding for a putative xylanase gene (named *XynA3*) was identified. The XynA3 ORF encodes a predicted protein of 371 amino acid residues, with a calculated molecular mass of 39,965 Da and a p*I* of 8.25. A potential ribosome-binding site (SD) sequence, 5′-AGGAGA-3′, was located 11 bp upstream of the translation start codon (ATG). BLASTX analysis revealed that the XynA3 displayed the highest identity (92%) with thermostable endo-1,4-xylanase from *Paenibacillaceae* bacterium JTherm (GenBank accession number: PDO11710) derived from the compost metagenome, and other endo-1,4-xylanases, members of glycosyl hydrolase family 11. Alignment of the amino acid sequence of XynA3 with those of other endo-β-1,4-xylanases showed presence of four conserved regions (black boxes highlighted in Fig. 4) and two conserved glutamic acid residues forming the catalytic site (green color highlighted in Fig. 4), typically observed in family 11 xylanases^28^. A highly-conserved asparagine (N) is found in position 73 (shown in bold in Fig. 4). The N is conserved in the alkaline xylanase group, and is responsible for the pH adaptation^28^.

### Search for XynA3 in sediment metagenomic dataset

A search of *XynA3* nucleotide sequence against the sequenced sediment metagenome revealed that only 8 reads, one short contig (414 bp) and one short ORF (87 bp) aligned to the *XynA3* sequence. This suggests that the organism that harbors this enzyme is present in this environment at low abundance. A higher sequencing coverage would be required to identify this enzyme using the sequence-based approach. Moreover, the identified short ORF that matched the *XynA3* sequence was not annotated as endo-1,4-β-xylanase in the CAZy database, most likely due to short sequence length or insufficient homology. Despite 23 putative xylanase sequences were identified using the sequence-based approach, the activity-based approach identified only one xylanase. The major drawback of the function-based screening is that the gene expression from uncultured organisms and proper folding of the expressed protein is not always achieved in *E. coli* host^49^. Both sequence- and function-based screening approaches therefore have their advantages and disadvantages. A combined analysis using both methods may be complementary and provide the better chance of novel enzyme discovery.

### Purification and biochemical characterization of XynA3

**Figure 5:**
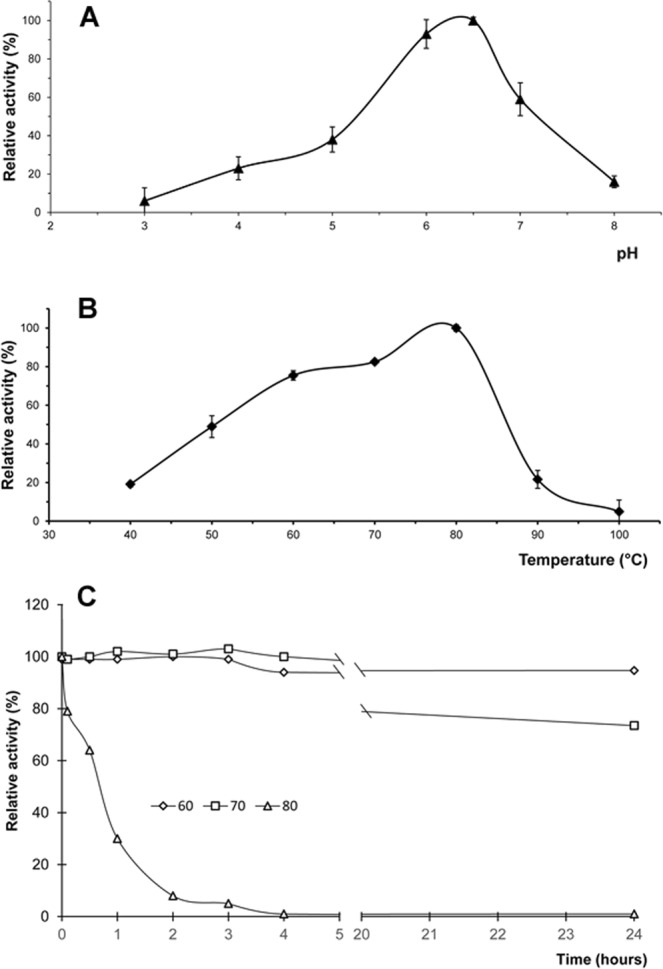
pH (**A**) and temperature (**B**) optima and thermostability (**C**) of XynA3 from *E*. *coli* (pET21a-xynA3). (**A**) Effect of pH on the activity. Enzyme activity was assayed in a pH range of 3.0–8.0. (**B**) Effect of temperature on the activity. Enzyme activity was assayed at various temperatures of 40–100 °C. (**C**) Thermal stability of purified XynA3 at pH 6.5 in the absence of xylan. Residual activity was monitored at various times (10 min, 30 min, 1 h, 2 h, 3 h, 4 h, and 24 h) after incubation at 60 °C (◊), 70 °C (□), and 80 °C (∆). The initial activity was defined as 100%.

Identified xylanase-encoding gene was cloned, expressed and characterized in *E*. *coli*. Recombinant enzyme was purified in two steps, Ni-NTA affinity chromatography and ion exchange chromatography. SDS-PAGE revealed that the purified protein showed single band with a molecular mass of 41 kDa (Fig. 3B), higher than the typical molecular mass of GH11 xylanases (<30 kDa)^50^. The molecular weight of the recombinant xylanases corresponds to the calculated molecular mass of the mature peptide, suggesting that the recombinant enzyme is not glycosylated. The specific activity of the enzyme preparation was 9080 U/mg on arabinoxylan and 5060 U/mg on beechwood xylan. There was no activity towards carboxymethyl cellulose (CMC) and cellulose (data not shown), indicating that the enzyme can be useful in paper production. The enzyme exhibited >80% of its maximal activity in the temperature ranging from 65 to 85 °C, with the highest activity recorded at 80 °C (Fig. 5A), and >80% of its maximal activity in the pH range of 5.5 to 6.8, with the highest activity at a pH of approximately 6.5 (Fig. 5B).

The xylanase was stable at temperature up to 70 °C. It maintained 94% of the original activity at 60 °C after incubation for 24 h and 73% of the original activity at 70 °C after incubation for 24 h (Fig. 5C). It lost its activity at temperature 80 °C after incubation for 4 h.

### The effect of metallic ions and inhibitors on the purified xylanase

At low concertation (1 mM), Fe^3+^ ions, dithiothreitol (DTT) and β-mercaptoethanol were found to increase the xylanase activity by 16%, 17%, and 144%, respectively. At high concertation (10 mM), β-mercaptoethanol increased the xylanase activity by 174%. Ca^2+^, K^+^ ions, EDTA, Tween 20 slightly inhibited xylanase activity by 9–19%, while Mn^2+^, Mg^2+^, Cu^+^, N-Bromosuccinimide, SDS and Tritone X-100 strongly inhibited its activity by 33–97% (Table 1). The presence of FeCl_3_ increased the activity while EDTA slightly inhibited the activity of the enzyme suggesting the enzyme is a metalloenzyme. Interestingly, the addition of β-mercaptoethanol enhanced the catalytic efficiency of the enzyme by 1.7-fold. The disulfide bonds reducing agents, β-mercaptoethanol and DTT, have been reported previously to stimulate activity of other xylanases and indicate that cysteine residues are a part of catalytic site in the xylanase^51^.

**Table 1:** Effect of different substances on relative activity (%) of purified XynA3 xylanase.

Substance	Concentration	
	1 mM	10 mM
Control	100	100
Ca^2+^	90	84
Fe^3+^	116	86
Mn^2+^	59	40
Mg^2+^	67	99
K^+^	86	93
Cu^+^	80	62
EDTA	93	81
DTT	117	72
2-Mercaptoethanol	244	274
N-Bromosuccinimide	91	3
**Substance**	**Concentration**	
	**0**.**1%**	**1%**
SDS	ND	40
Tween 20	94	91
Tritone X-100	12	44

### Production of xylose and xylooligosaccharides by purified xylanase

**Figure 6:**
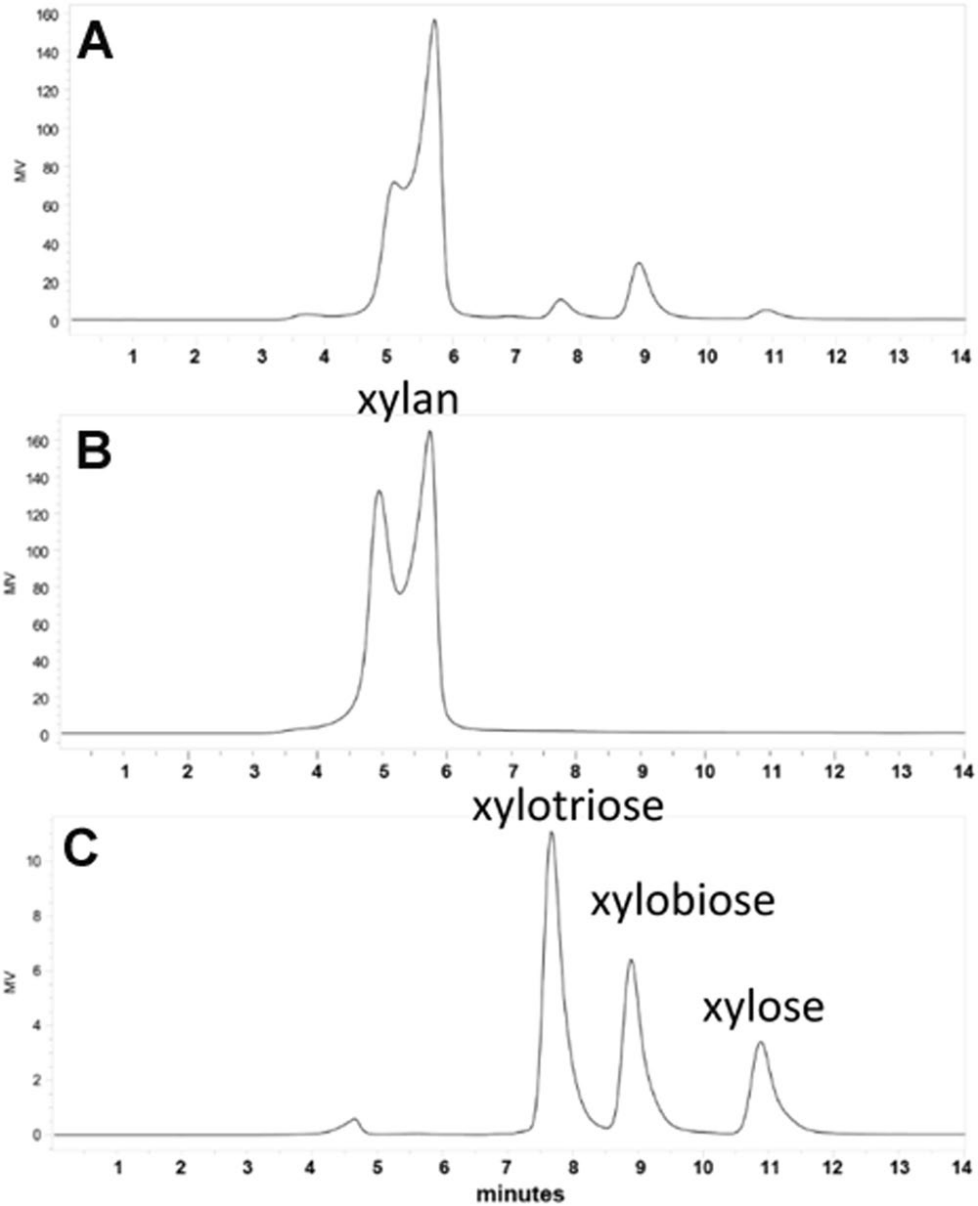
HPLC analysis of xylooligosaccharides produced by XynA3 enzyme from Beechwood xylan. (**A**) Products released after incubation of 2% Beechwood xylan with XynA3. (**B**) Beechwood xylan (substrate). (**C**) Xylose, xylobiose and xylotriose (standards).

HPLC analysis revealed that the recombinant xylanase released mainly xylobiose and to a lower extent of xylotriose and xylose as main products of beechwood xylan hydrolysis (Fig. 6). This characteristic is observed in GH11 xylanases, which produce xylobiose and xylotriose as main end reaction products from xylans^52^.

### Protein model analysis

**Figure 7:**
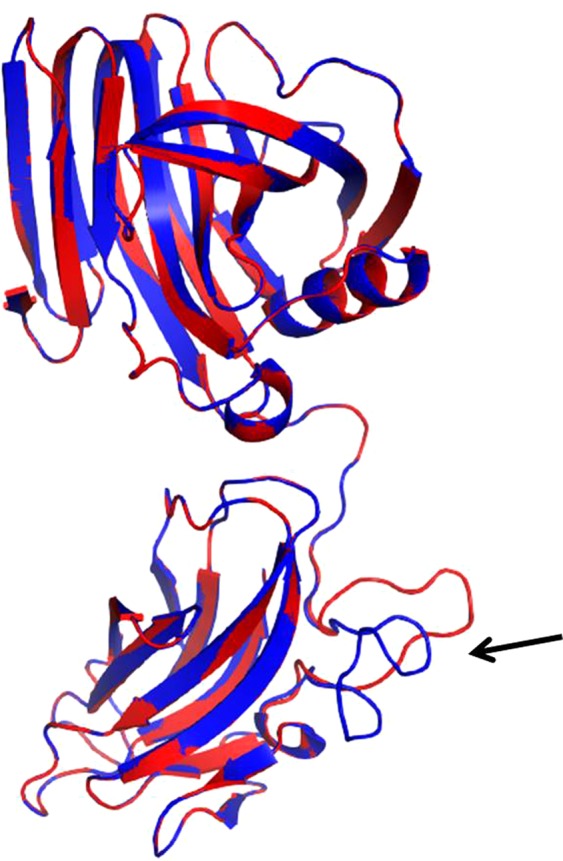
Ribbon representation. Ribbon diagram corresponding to the prediction of the tertiary structure of XynA3 (blue) using the Swiss-Model program superimposed with the prediction of the tertiary structure of the 1,4-beta-xylanase from *P*. *bacterium* JTherm (red). The black arrow shows the linker sequence between domains.

A prediction of the tertiary structure of the XynA3 and the 1,4-beta-xylanase from *Paenibacillaceae* bacterium JTherm was made using Swiss-Model^32^. In both cases, the structure of the alkaliphilic XynJ from *Bacillus* sp. 41M-1 (Protein Data Bank code 2dcj.1.A) was used as a template to build the models. XynA3 and the 1,4-beta-xylanase from *P*. *bacterium* JTherm showed 74.7% and 75.0% sequence identity, respectively, with the xylanase XynJ. Both proteins, XynA3 and the 1,4-beta-xylanase from *P*. *bacterium* JTherm, showed a similar structure, with two well-differentiated domains: a glycoside hydrolase (GH) family 11 catalytic domain at the N-terminus and a carbohydrate binding module (CBM) of the family 6 at the C-terminus. Joining the two domains there is a linker sequence that presents differences in both proteins (Fig. 7). This linker sequence protrudes in opposite directions in both models and corresponds to a GSG insertion in the XynA3 sequence not present in the 1,4-beta-xylanase from *P*. *bacterium* JTherm. It is an area rich in small and polar amino acids such as G, S and T (GGSGGGSGGGTST) that is characteristic of flexible linkers connecting domains that require a certain degree of movement or interaction^53^. Although it is difficult to predict its importance at a biological level, it could facilitate the affinity or selection of certain ligands for the CBM domain and could determine the difference between the two proteins.

## Conclusions

Metagenomic sequencing of the DNA extracted from the Lobios hot spring sediment (Ourense, Spain) demonstrated the predominance of Bacteria (93%) over Archaea (7%), being Acidobacteria the most abundant phylum. Sequence-based analysis showed that the hot spring metagenome is a potential source of novel microbial xylanases. A novel GH11 family xylanase, XynA3, was isolated by screening of the fosmid metagenomic library. The purified enzyme is low molecular weight (41 kDa), cellulase-free, thermostable (stability at high temperature of 60–70 °C), active at mildly acidic pH (pH 5.5 to 6.8) and producing xylobiose and xylotriose, suggesting that the enzyme is potentially useful for various industrial purposes, for example for pulp bleaching process or prebiotics production.

## Supplementary information

Dataset 1
